# Aging Effects of *Caenorhabditis elegans* Ryanodine Receptor Variants Corresponding to Human Myopathic Mutations

**DOI:** 10.1534/g3.117.040535

**Published:** 2017-03-21

**Authors:** Katie Nicoll Baines, Célia Ferreira, Philip M. Hopkins, Marie-Anne Shaw, Ian A. Hope

**Affiliations:** * Leeds Institute of Biomedical and Clinical Sciences, St James’s University Hospital, LS9 7TF, United Kingdom; † School of Biology, Faculty of Biological Sciences, University of Leeds, LS2 9JT, United Kingdom

**Keywords:** *Caenorhabditis elegans*, aging, ryanodine receptor, malignant hyperthermia, muscle

## Abstract

Delaying the decline in skeletal muscle function will be critical to better maintenance of an active lifestyle in old age. The skeletal muscle ryanodine receptor, the major intracellular membrane channel through which calcium ions pass to elicit muscle contraction, is central to calcium ion balance and is hypothesized to be a significant factor for age-related decline in muscle function. The nematode *Caenorhabditis elegans* is a key model system for the study of human aging, and strains were generated with modified *C. elegans* ryanodine receptors corresponding to human myopathic variants linked with malignant hyperthermia and related conditions. The altered response of these strains to pharmacological agents reflected results of human diagnostic tests for individuals with these pathogenic variants. Involvement of nerve cells in the *C. elegans* responses may relate to rare medical symptoms concerning the central nervous system that have been associated with ryanodine receptor variants. These single amino acid modifications in *C. elegans* also conferred a reduction in lifespan and an accelerated decline in muscle integrity with age, supporting the significance of ryanodine receptor function for human aging.

To improve the health of the world’s aging population we need a better understanding of the aging processes, and age-related decline of skeletal muscle function is of key importance. Defective excitation-contraction coupling (Payne and Delbono 2004) and reduced capacity of Ca^2+^ homeostasis (Weisleder *et al.* 2006; Zhao *et al.* 2008) have been suggested to contribute to the human muscle contractile dysfunction that occurs with age. The ryanodine receptor isoform 1 (RyR1) is the channel through which Ca^2+^ is released from the skeletal muscle sarcoplasmic reticulum to elicit contraction. In the mouse there is an age-related increase in the ryanodine receptor “leakiness” (Anderson *et al.* 2011) and age-related decrease in both the number of RyR1s and their degree of coupling to regulatory proteins (Ryan *et al.* 2000). Single-point variants in the human *RYR1* gene have been associated with the impairment of calcium handling in malignant hyperthermia (MH) (Robinson *et al.* 2002, 2006; Bouchama and Knochel 2002; McCarthy *et al.* 2000; Tong *et al.* 1997; Jungbluth *et al.* 2009; Loseth *et al.* 2013; Nishio *et al.* 2009). The clinical incidence of MH is age-dependent and there is evidence of premature aging in MH mouse models (Boncompagni *et al.* 2006, 2009). During an MH episode, the sensitized RyR1 is activated by inhalational anesthetics and remains open without neural stimulation, resulting in sustained muscle contraction across the body (Larach *et al.* 1994), with death in the absence of a prompt and aggressive treatment regimen. The primary method of diagnosing susceptibility to this condition is through an *in vitro* contracture test (IVCT) (Ording *et al.* 1997), which measures the response of patients’ muscle biopsy specimens to the inhalation anesthetic halothane and to caffeine.

Mammalian RyR1 is a very large tetrameric membrane protein (>5000 amino acid residues per monomer) (Robinson *et al.* 2006), making it difficult to study. Similarly, the human *RYR1* gene, with its many introns across such a large coding region, is awkward to manipulate. The nematode *Caenorhabditis elegans*, however, has a compact genome and the *RYR1* ortholog, *unc-68*, is only 30 kb (WormBase). Nevertheless, UNC-68 has ∼40% amino acid identity with the human RyR1, distributed along the entire length of the proteins, suggesting that the mammalian and nematode ryanodine receptor operate and are controlled in similar fashion (Sakube *et al.* 1997). The short lifespan and many other attributes of this species make *C. elegans* the ideal subject for investigating the contribution of human RyR1 variants to aging.

## Materials and Methods

### Recombineering

Amino acid sequence alignment identified residues of RyR1 which were variant in human genetic conditions but conserved in *C. elegans* UNC-68 (Table 1). Modification of the target gene, *unc-68*, was achieved by a two-step counterselection recombineering technique (Feng *et al.* 2012). A PCR-amplified variant-specific counterselection cassette was inserted into the target fosmid (WRM069cA02) by bacterial transformation, using positive selection for the cassette. The cassette was then replaced, with incorporation of the desired point mutation in a second bacterial transformation with a second PCR product bearing the desired sequence alteration, using negative selection against the cassette. A dicistronic cassette was used containing the positive marker *tet*A(C) conferring tetracycline resistance (Tc^R^), and the negative marker *rps*L^+^ conferring streptomycin sensitivity (Str^S^) in the *rps*L^−^ (thus Str^R^) EL350 host, a recombineering competent *Escherichia coli* strain (Feng *et al.* 2012). Confirmation of insertion and replacement of the cassette was carried out using colony PCR. The final recombineered fosmid for each variant was sequenced across the manipulated region and subjected to *Eco*RI restriction enzyme digestion to confirm that the correct variant had been introduced into the fosmid, with at least no substantial rearrangements.

**Table 1:** Amino acid alignment in region of all eight variants studied and the corresponding transgenic *C. elegans* strains generated

Human Condition	*RyR1* Variant	Protein	Alignment	Transgenic Strain with Variant Fosmid Only	Transgenic Strain Also Including Wild-Type Fosmid
Malignant hyperthermia	p.G341R c.1021G > A	RyR1	KRDV**EGMG**PPE**I**K**YGE**SLC**F**V**QHV**ASG**LW**	UL4141 (UL4193)	UL4167 (UL4200)
		UNC-68	EKEE**EGMG**NAT**I**R**YGE**TNA**F**I**QHV**KTQ**LW**		
	p.R2163H c.6488G > A	RyR1	**D**TMSL**L**EC**L**G**QIR**S**LL**I**VQ**MGPQ**EE**NLMI	UL4147 (UL4194)	UL4153 (UL4198)
		UNC-68	**D**VTDF**L**VY**L**I**QIR**E**LL**T**VQ**FEHT**EE**AILK		
	p.R2454H c.7361G > A	RyR1	**CAP**EMHL**IQAGKG**EA**LR**I**RAILRSL**VP**L**E	UL4143 (UL4195)	UL4165 (UL4206)
		UNC-68	**CAP**DPMA**IQAGKG**DS**LR**A**RAILRSL**IS**L**D		
	p.R2458H c.7373G > A	RyR1	**IQAGKG**EA**LR**I**RAILRSL**VP**L**E**DL**VG**I**IS	UL4144 (UL4201)	UL4158 (UL4197)
		UNC-68	**IQAGKG**DS**LR**A**RAILRSL**IS**L**D**DL**GQ**I**LA		
Central core disease	p.R4861H 14582G > A	RyR1	**VVVYLYTV**V**AFNFFRKFY**-NKS**E**DED**EPD**	_	UL4152 (UL4205)
		UNC-68	**VVVYLYTV**I**AFNFFRKFY**VQEG**E**EGE**EPD**		
	p.A4940T c.14820G > A	RyR1	**FFFFVI**V**ILLAI**I**QGLIIDAFGELRDQQE**	UL4157 (UL4203)	UL4156 (UL4202)
		UNC-68	**FFFFVI**I**ILLAI**M**QGLIIDAFGELRDQQE**		
Exertional heat illness	p.R163C c.487C > T	RyR1	**CWWT**M**HPASKQRSEGEKVRVGDD**I**ILVSV**	UL4155 (UL4191)	UL4160 (UL4192)
		UNC-68	**CWWT**I**HPASKQRSEGEKVRVGDD**V**ILVSV**		
Late-onset axial myopathy	p.K3452Q c.10354A > C	RyR1	**IF**IY**WS**K**S**HN**FKREE**Q**N**F**V**V**Q**N**E**INNMSF	UL4168 (UL4196)	UL4169 (UL4199)
		UNC-68	**IF**RI**WS**Q**S**QH**FKREE**L**N**Y**V**A**Q**F**E**EDAAAT		

Human variant residues and corresponding *C. elegans* residues are underlined. Amino acids identical in RyR1 and UNC-68 are in bold. Strains in brackets also have the chromosomally integrated *myo-3*::*gfp*, *rol-6(su1006)* transgenes. UL4140 is transgenic for the wild-type *unc-68* fosmid and UL4190 is the corresponding strain with the chromosomally integrated *myo-3*::*gfp*, *rol-6(su1006)* transgenes.

### Strains

Manipulated and wild-type *unc-68* fosmids were introduced into *unc-68(e540)* worms by microinjection (Mello *et al.* 1991). *unc-68(e540)* carries a point mutation toward the center of the gene and behaves genetically as a null (Maryon *et al.* 1996). Those worms bearing the fosmid in an extrachromosomal array encoding a functional *unc-68* display a wild-type phenotype of movement through which they could be selected and transgenic strains established. One fosmid was also coinjected in a mixture with pRF4, a plasmid bearing *rol-6(su1006)*, which causes an obvious dominant roller phenotype (Mello *et al.* 1991).

GFP-myosin strains were developed by mating N2 males with *unc-68(e540)* hermaphrodites to generate male progeny heterozygous for *unc-68*. These males were mated with RW1596 {(stEx30 [*myo-3*::*gfp*, *rol-6(su1006)*]}. The resulting hermaphrodite progeny were then allowed to self-fertilize to generate uncoordinated rollers, homozygous for *unc-68(e540)* and bearing the extrachromosomal array containing *myo-3*::*gfp*, *rol-6(su1006)*. These worms were subjected to UV mutagenesis and screened for uncoordinated rollers with progeny that are all rollers due to the extrachromosomal array having been stably integrated into a chromosome (Mariol *et al.* 2013). Hermaphrodites from this new strain were mated with males from each of the *unc-68* fosmid transgenic strains screening for worms with well-coordinated roller movement due to the *unc-68* bearing extrachromosomal array and the chromosomally integrated *myo-3*::*gfp*, *rol-6(su1006)* transgenes. Self-fertilization and selection for well-coordinated rollers yielded *myo-3*::*gfp*, *rol-6(su1006)* homozygous strains bearing the *unc-68* transgenes.

### Strain maintenance

All *C. elegans* strains were routinely maintained in culture at 20° on 50 mm plates of Nematode Growth Medium (NGM) (51 mM NaCl, 1.7% agar, 0.25% Bacto-peptone in 1 liter H_2_O, autoclaved, before addition of 1 ml 1 M CaCl_2_, 1 ml 1 M MgSO_4_, 25 ml 1 M KPO_4_ pH 6.0, 1 ml cholesterol 5 mg/ml in ethanol) (Stiernagle 2006). NGM plates were seeded with *E. coli* strain OP50. Transgenic strains were maintained by serial transfer of transgenic worms selected for their altered phenotype.

### Age synchronizing

Eggs were prepared by bleaching to synchronize worm populations for assay. Mixed stage population of nematodes were washed from the NGM plates in 500 μl M9 buffer (20 mM KH_2_PO_4_, 42 mM Na_2_HPO_4_, 86 mM NaCl, 1 mM MgSO_4_) (Stiernagle 2006). A total of 150 μl Sainsbury’s thin bleach and 100 μl 4 M NaOH were added and the solution left at room temperature for 5 min. After microcentrifugation at 13,500 rpm for 30 sec the supernatant was removed and the pellet resuspended in 1 ml fresh M9 buffer. Centrifugation was repeated and the pellet resuspended in ∼50 μl residual supernatant for transfer to a freshly seeded NGM plate. This protocol kills all postembryonic stages leaving the eggs, which subsequently hatch across the 14 hr of embryogenesis, and then develop together into adults, effectively ensuring that the worms assayed will be of approximately the same age in days.

### Phenotyping assays

The transgenic strains were assayed to determine their sensitivity to caffeine and halothane. Individual adult worms 4 d after synchronization were selected from NGM plates using a sterile worm pick and placed in 1 ml of 0, 1, 5, 10, 20, 40, or 80 mM caffeine dissolved in S-medium {1 liter S Basal [5.85 g NaCl, 1 g K_2_HPO_4_, 6 g KH_2_PO_4_, 1 ml cholesterol (5 mg/ml in ethanol), H_2_O to 1 liter and autoclaved], 10 ml 1 M potassium citrate pH 6, 10 ml trace metals solution [1 liter stock: 1.86 g Na_2_ EDTA, 0.69 g FeSO_4_•7H_2_O, 0.2 g MnCl_2_•4H_2_O, 0.29 g ZnSO_4_•7H_2_O, 0.025 g CuSO_4_•5H_2_O, H_2_O to 1 liter, autoclaved, and stored in the dark], 3 ml 1 M CaCl_2_, 3 ml 1 M MgSO_4_} (Stiernagle 2006). After 5 min, the effect of the chemical was quantified by assessing the number of body bends in 30 sec. Halothane assays were carried out in a similar manner but using 1 ml of 0, 0.5, 1, 1.5, 2, and 2.5 mM halothane solution (prepared from a 25 mM stock in DMSO and mixed into S-medium) and assaying after 1 min of exposure. Fifty worms were assayed for each strain at each concentration for each reagent.

### RNAi assays

Synchronized L1s were transferred to new NGM plates, including 1 mM IPTG and 50 μg/ml ampicillin, and seeded with *E. coli* (HT115) producing dsRNA for *cbd-1*, *osm-3*, or *che-3. cbd-1* RNAi was used in the longevity assays to exclude progeny from the assay (Johnston *et al.* 2010). *cbd-1* is only required for eggshell production and *cbd-1* RNAi knockdown appears to have no effect on longevity of hatched animals. Lifespan assays were initiated at the young adult stage and populations were scored every day. Animals that were lost from the plates or died from vulval extrusion were excluded from the analysis. *osm-3* and *che-3* RNAi–treated animals were used in caffeine assays as described above, with controls treated identically except using HT115 containing L4440 RNAi plasmid lacking an insert so that no dsRNA was present.

### Muscle aging assays

The transgenic strains expressing the *myo-3*::*gfp* were assayed on days 0, 2, 4, 6, 8, 10, 12, and 14 of adulthood. Day 0 was considered to be 3 d posthatching. Only live worms were selected for analysis. A total of 20 worms were assayed for each strain at each time point. The extent of muscle aging was quantified by direct observation using an aging scale of 1–5 (Figure 4). A score of 1 indicates perfectly ordered myofilament structure and a score of 5 indicates total disorder, with half scores for worms that lay between the defining states. Visualization and image capture was carried out using a Leica DMR fluorescence microscope and Improvision Openlab software. In preparation for microscopy, animals were immobilized using 5 mM levamisole and placed in individual wells of an eight-well microscope slide. Each individual was scored for extent of muscle aging at the head, vulva, and tail regions of the body at 20× magnification. These scores were combined to provide a whole-body score.

### Statistical analysis

Results of phenotyping assays were analyzed to establish any potential differences in the rate of body bends when the worms were subjected to halothane and caffeine. Each strain containing an altered fosmid was compared to the strain containing the unaltered fosmid at each discrete concentration of the reagent in question. A linear model was established describing body bends being dependent upon presence of the variant, and statistical significance was measured by carrying out one-way ANOVA on the linear model. Body bends data on RNAi-treated worms were also compared by one-way ANOVA at each discrete concentration to determine whether there was a significant difference in movement from a mock RNAi treatment with the L4440 empty plasmid RNAi control.

Categorical whole-body muscle score data were analyzed using ordered logistic regression, with *P* values calculated by comparing the T-statistic to the standard normal distribution. This enabled examination of any differences between the strains with modified *unc-68* and the strains rescued with the wild-type *unc-68*, evaluation of the effect of increasing age of the worm in days, and interactions between these two variables. Differences between the scores for the regions of the worm down the anterior-posterior axis were similarly carried out. All statistical analyses were completed using RStudio version 3.0.2.

Comparison of lifespan data were subjected to survival analysis with curve comparison, using the log-rank test to determine significant differences between variant strains and the UL4140 wild-type control, as well as testing for any difference between the UL4140 wild-type control and N2.

### Reagent and data availability

Strains and recombineered fosmids described are available upon request. Supplemental Material, File S1 contains the raw data for the experiments presented.

## Results and Discussion

### A single fosmid DNA clone contains the whole of unc-68

UNC-68, and release of Ca^2+^ from the sarcoplasmic reticulum, is needed for wild-type locomotion. *C. elegans* locomotion is achieved through the coordinated contraction and relaxation of opposing dorsal and ventral muscle cells, resulting in a sinusoidal wave passing along the body, propelling the worm forward or backward (Nicholas 1984). While mouse mutants that lack RyR1 are not viable, *unc-68* null mutants survive (Sakube *et al.* 1997). The relatively small size of the nematode’s muscle cells means influx of calcium through the cell membrane alone is sufficient for muscle contraction. However, muscle contractions of the *unc-68* mutant are not as strong or rapid as in the wild-type, and consequently, the overall appearance of locomotion is affected (Sakube *et al.* 1997). Microinjection transformation of *unc-68* mutant hermaphrodites with genomic DNA fosmid clone WRM069cA02 (WormBase) yielded progeny with apparently wild-type locomotion, suggesting WRM069cA02 contains the entire *unc-68* gene and all that is required for its expression. Each rescued line moved with ∼200 body bends per min in liquid medium, considerably faster than the 60 mean body bends per min of the *unc-68* null mutant. Transmission of this rescued phenotype to subsequent generations established that this fosmid, in an extrachromosomal transgenic array, provides appropriate levels of expression of the ryanodine receptor and yielded the reference strain, UL4140, used in subsequent comparisons examining ryanodine receptor variants.

**Figure 1:**
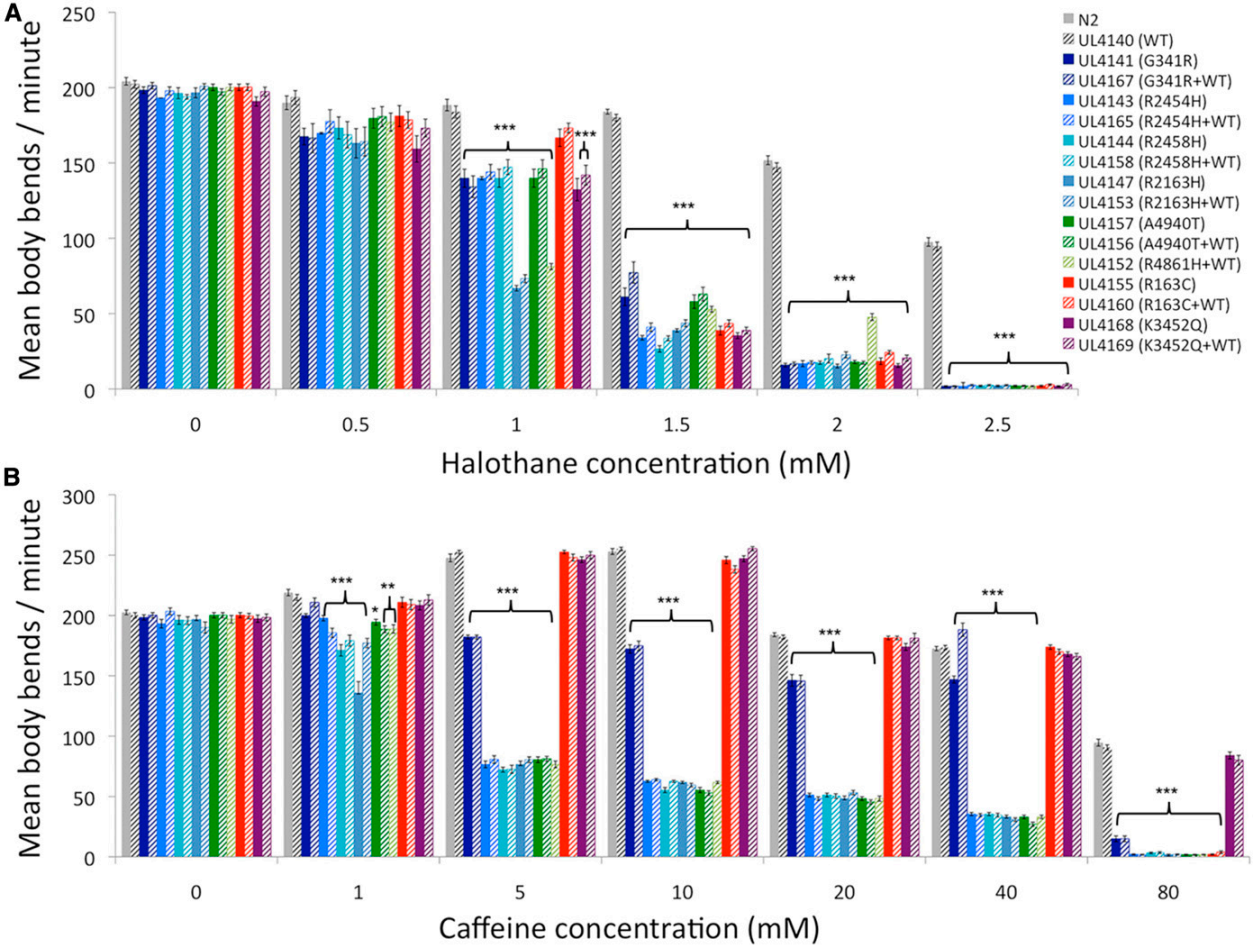
Comparison of the rate of locomotion of *unc-68* variant strains and wild-type *C. elegans* in increasing concentrations of halothane or caffeine. Mean body bends per minute for 50 individuals in the presence of various concentrations of halothane (A) or caffeine (B) are presented for each strain. *C. elegans* strains corresponding to malignant hyperthermia (MH)-associated variants are in shades of blue, central core disease (CCD)-associated variants are in shades of green, exertional heat illness (EHI)-associated variant is in red, and late-onset axial myopathy (LOAM)-associated variant is in purple. In the key, strain names are provided, with the nature of the RyR1 variant and whether the wild-type fosmid (WT) was also present indicated in brackets. Transgenic strains generated using only a variant fosmid are represented by solid bars, while strains generated using a variant fosmid and the wild-type fosmid are represented by striped bars, in adjacent corresponding pairs. The solid gray bars are for the wild-type N2 while the dashed gray bars are for the control transgenic strain, UL4140, generated with just the wild-type *unc-68*. Error bars are SEM. Significant differences between variant strains and UL4140 are indicated: * *P* < 0.05, ** *P* < 0.01, *** *P* < 0.001 (one-way ANOVA).

Not only did the wild-type *unc-68* transgene in UL4140 fully rescue for locomotion but the strain responded to increasing concentrations of halothane and caffeine, the drugs used in the MH IVCT, in the same manner as the standard wild-type strain, N2 (Figure 1). *C. elegans* responds to inhalation anesthetics such as halothane in a similar manner to humans; initial excitation leads on to lack of coordination, before complete cessation of movement, first with and then without response to mechanical stimulation, with longer term exposure being fatal (Morgan and Carscobi 1985). Halothane inhibited UL4140 and N2 locomotion progressively and to the same degree with increasing concentrations of the anesthetic, across concentrations tested (0.5–2.5 mM) (Figure 1A). Previous work showed *unc-68* mutants to have altered responses to caffeine (Adachi and Kagawa 2003), yet locomotion of both UL4140 and N2 individuals was stimulated and then inhibited to the same degree by increasing concentrations of caffeine (from 1 to 80 mM) (Figure 1B).

### UNC-68 variants equivalent to RyR1 myopathic variants retained ryanodine receptor function

Eight human *RYR1* variants were selected for study in *C. elegans* (Table 1). Selection of variants took into account that some *RYR1* variants are implicated in other myopathic conditions beyond MH: central core disease (CCD) (Robinson *et al.* 2002), exertional heat illness (EHI) (Davis *et al.* 2002; Nishio *et al.* 2009), and late-onset axial myopathy (LOAM) (Jungbluth *et al.* 2009; Loseth *et al.* 2013). CCD is a congenital myopathy that presents with progressive proximal muscle weakness: type 1 skeletal muscle fibers exhibit cores with unstructured myofibrils lacking mitochondria (Zhou *et al.* 2007). In EHI, individuals suffer potentially lethal hyperthermic responses to exercise. Patients with LOAM exhibit lumbar hyperlordosis, scapular winging, and camptocormia due to skeletal muscle defects, with onset between the ages of 30 and 70 yr (Jungbluth *et al.* 2009; Loseth *et al.* 2013). Strikingly, the amino acid residues variant in the different conditions are not segregated to distinct domains of the ryanodine receptor and are distributed throughout the protein (Robinson *et al.* 2006).

The RyR1 variants selected for study were: G341R, R2163H, R2454H, and R2458H for MH (Robinson *et al.* 2006); R4861H implicated in CCD only (Monnier *et al.* 2001); A4940T implicated in CCD and MH (Kraeva *et al.* 2013); R163C implicated in EHI, CCD, and MH (Estève *et al.* 2010); and K3452Q implicated in LOAM (Jungbluth *et al.* 2009; Loseth *et al.* 2013). Single base pair changes were generated by recombineering of the *unc-68* fosmid WRM069cA02 such that the change in the encoded UNC-68 would be precisely equivalent to the single amino acid changes in these RyR1 variants.

Multiple strains were generated by microinjection transformation of the *unc-68* mutant with each modified fosmid. All the variants constructed, apart from that for R4861H, rescued the mutant phenotype. Therefore, the identity of these particular amino acid residues, with the one exception, is not critical for basic ryanodine receptor function under normal conditions, despite their evolutionary conservation from humans to nematodes. The progeny of *unc-68* null mutants microinjected with the CCD-associated R4861H variant all appeared to retain the *unc-68* phenotype, and transgenic progeny could not be distinguished from their nontransgenic siblings. Coinjection of the fosmid for the R4861H variant, along with a distinct transformation marker, yielded transgenic worms recognizable from the roller phenotype but still with a slow moving *unc-68* mutant phenotype, suggesting that the modification equivalent to R4861H had indeed inactivated, or at least severely compromised, UNC-68 function. The rolling phenotype means a numeric comparison of body bends would not be meaningful. No human individuals homozygous for R4861H are known and this amino acid substitution may inactivate the human ryanodine receptor too.

### Increased halothane sensitivity was conferred by the UNC-68 variants

All the rescued strains established with the modified *unc-68* fosmids exhibited an increased sensitivity to halothane (Figure 1A, solid bars) revealing that these single amino acid changes did modify the function of the ryanodine receptor and conferred an altered phenotype. An ∼10% decrease in the rate of body bends in liquid, in comparison to the control strains with wild-type *unc-68*, was apparent even at 0.5 mM halothane. At 1.5 mM halothane, a concentration that has little effect on the wild type, the rate of movement in the variant strains was reduced to 15% of that in the absence of halothane. The variant strains were completely paralyzed by 2.5 mM halothane, while the wild-type strains retained a reduced but still substantial mobility. Previous extensive investigations identified genes such as *unc-79*, *unc-80*, and *gas-1* from mutations conferring halothane hypersensitivity in *C. elegans* (Kayser *et al.* 1999; Sedensky and Meneely 1987). Mutations in *unc-68* may not have been isolated in these studies due to the need for specific point mutations that sensitize but do not eliminate ryanodine receptor function.

### Increased caffeine sensitivity was conferred by some of the UNC-68 variants

Young adults of the variant strains generated specific to MH or CCD also demonstrated a modified response to caffeine across the range of concentrations assayed, while those for EHI and LOAM did not (Figure 1B). The strains generated for the human *RYR1* variants G341R, R2454H, R2458H, R2163H, A4940T, and R4861H all failed to show the stimulation of locomotion of the wild type as caffeine was increased from 1 to 10 mM. These same strains, with the exception of that for variant G341R, actually displayed a substantial inhibition of locomotion from 1 to 5 mM caffeine, with a small progressive further inhibition from 10 to 40 mM, and almost complete paralysis at 80 mM, a concentration at which the wild type still remains motile. The G341R variant strain only shows a substantial inhibition of locomotion upon raising the caffeine concentration to 80 mM, but this is still not quite to the same degree as the other variant strains. The LOAM-associated K3452Q variant strains showed a wild type response to all concentrations of caffeine assayed, as did the EHI-associated R163C variant strain with the exception of almost total inhibition of locomotion specifically at the highest concentration of 80 mM.

### The modified unc-68s showed genetic dominance

Genetic dominance, a striking property of many pathogenic human *RYR1* variants and all those selected for study, was also demonstrated by the equivalent versions of *unc-68*. This was revealed when transgenic strains of *C. elegans* were generated by microinjection transformation using the modified *unc-68* fosmids but mixed with the unmodified fosmid, containing wild-type *unc-68* (Figure 1, striped bars). These strains all behaved in essentially the same manner as the strains transformed with only the corresponding modified *unc-68* across the range of caffeine and halothane concentrations assayed: the variant UNC-68s that conferred a modified sensitivity to caffeine or halothane did so even when wild-type UNC-68 was also present. Remarkably, when wild-type UNC-68 was also present, the *unc-68* variant for R4861H, the one, apparently nonfunctional, variant unable to rescue the *unc-68* mutant, nevertheless induced the same modified response to caffeine and halothane (and the same aging effects, see the next section) as other MH- and CCD-associated versions of UNC-68, like the corresponding human condition. This could result from the nonfunctional variant being stably expressed and, in combination with the wild-type protein, generating a malfunctioning heteromeric ryanodine receptor.

### The modified unc-68s conferred age-related phenotypes

**Figure 2:**
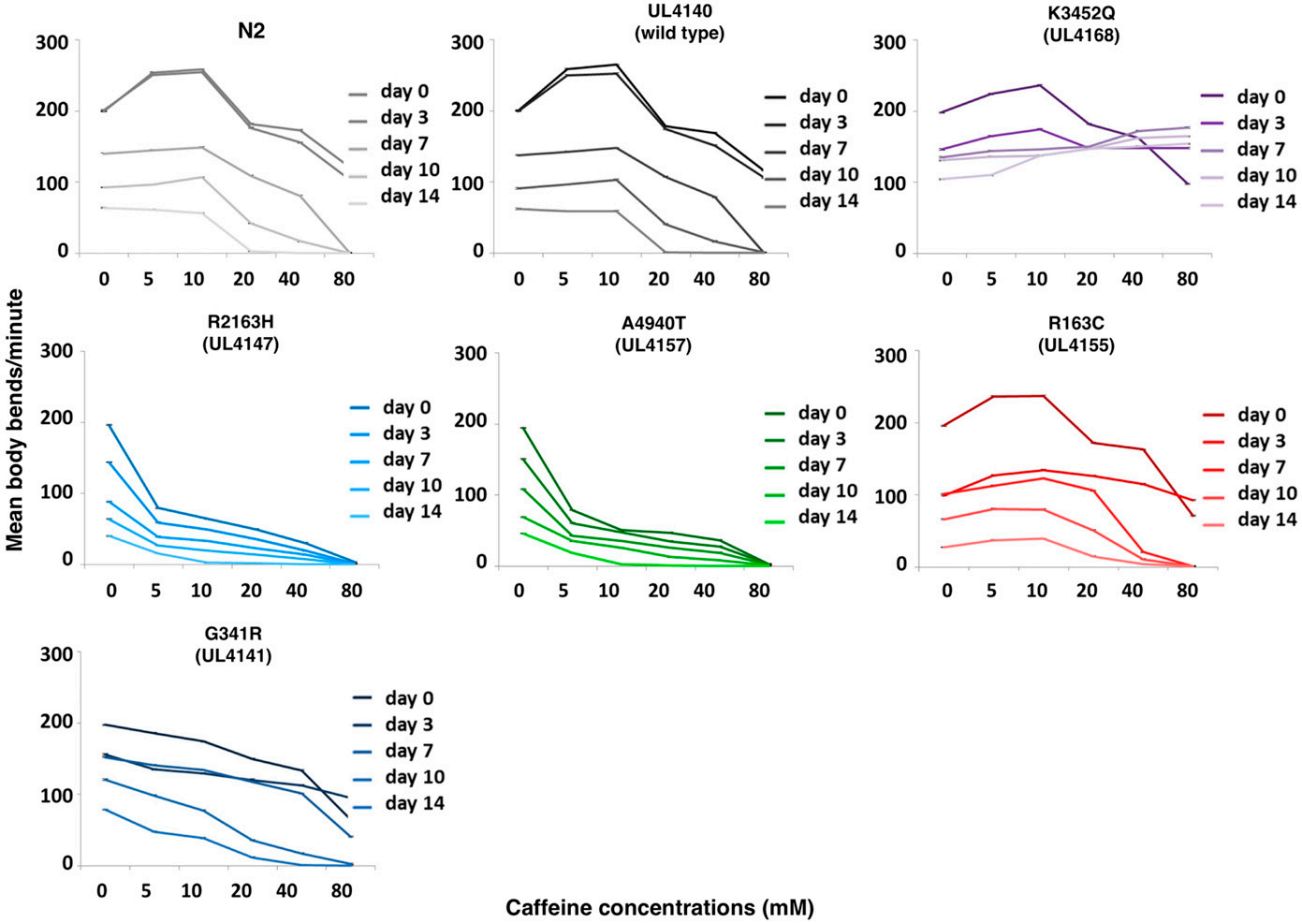
Locomotion of *C. elegans* expressing the LOAM associated variant of *unc-68* is specifically increasingly stimulated by caffeine with age. The locomotion of the strain for the RyR1 variants G341R (UL4141), R2163H (UL4147), A4940T (UL4157), R163C (UL4155), and K3452Q (UL4168) are compared with the strain transgenic for only the wild-type *unc-68* (UL4140) and the standard wild-type strain (N2). Mean body bends per minute in the presence of increasing concentrations of caffeine are presented for 50 individuals at 0, 3, 7, 10, and 14 d of adulthood. The color coding from Figure 1 is retained with, broadly, blue for MH, green for CCD, red for EHI, and purple for LOAM. Error bars are SEM.

Given the potential links between RyR1 variants and age-related phenotypes in mammals, the *unc-68* variant strains were examined for age-related phenotypes in *C. elegans*. First, a dramatic age-related change in response to caffeine was found for the LOAM (K3452Q) variant strain (Figure 2). Young adults of the LOAM variant strain showed the same response to caffeine as the wild type, with stimulation of locomotion at low concentrations and inhibition at higher concentrations. All the other strains, *i.e.*, those with other *unc-68* variants, showed simply a progressive dampening of the rate of locomotion with age but the same general response profile, with increasing concentrations of caffeine, characteristic of each strain (Figure 2). In contrast, rather than an inhibition of locomotion, older LOAM variant strain adults showed a considerable stimulation of locomotion, increasing with increasing caffeine concentrations, a response attained progressively as the animals aged. It is tempting to link this striking effect in *C. elegans* directly to the specific age-related symptoms characteristic of LOAM.

**Figure 3:**
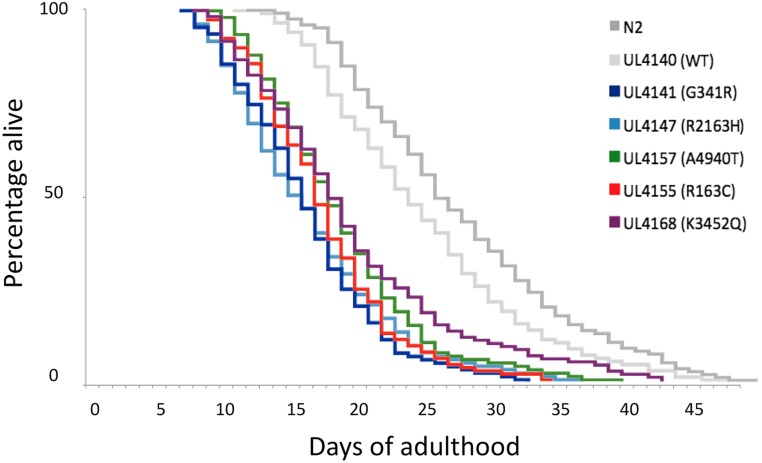
Single amino acid changes in UNC-68 shorten *C. elegans* lifespan. The percentage of animals surviving on successive days of adulthood is presented for each strain: strains transgenic for different *unc-68* variants; UL4140, transgenic for just the wild-type *unc-68* (light gray); and the nontransgenic wild-type N2 (dark gray). In the key, strain names are provided, with the nature of the RyR1 variant indicated in brackets. The color coding from Figure 1 is retained with, broadly, blue for MH, green for CCD, red for EHI, and purple for LOAM.

Second, the *unc-68* variants caused a decrease of median lifespan (Figure 3). All the *unc-68* variant transformed strains that were assayed had median lifespans of 14–17 d into adulthood, compared to the 22 or 24 d for the wild-type control strains. A log-rank test on the survival curves revealed that lifespans of strains transgenic for variant UNC-68s were statistically shorter than the lifespan of UL4140, the control strain transgenic for the wild-type UNC-68 (*P* < 0.0001 in each case, File S1). Hence although the ryanodine receptor appeared to remain functional, these single amino acid changes did shorten *C. elegans* lifespan.

It is also noted that the lifespan of UL4140 is significantly shorter than the lifespan of the nontransgenic wild-type control, N2, although the difference is not as marked (*P* < 0.05, File S1). Presumably this small reduction in lifespan results from the difference in expression of *unc-68* when present in multiple copies as an extrachromosomal transgene, rather than in the endogenous location in single copy, within a chromosome.

**Figure 4:**
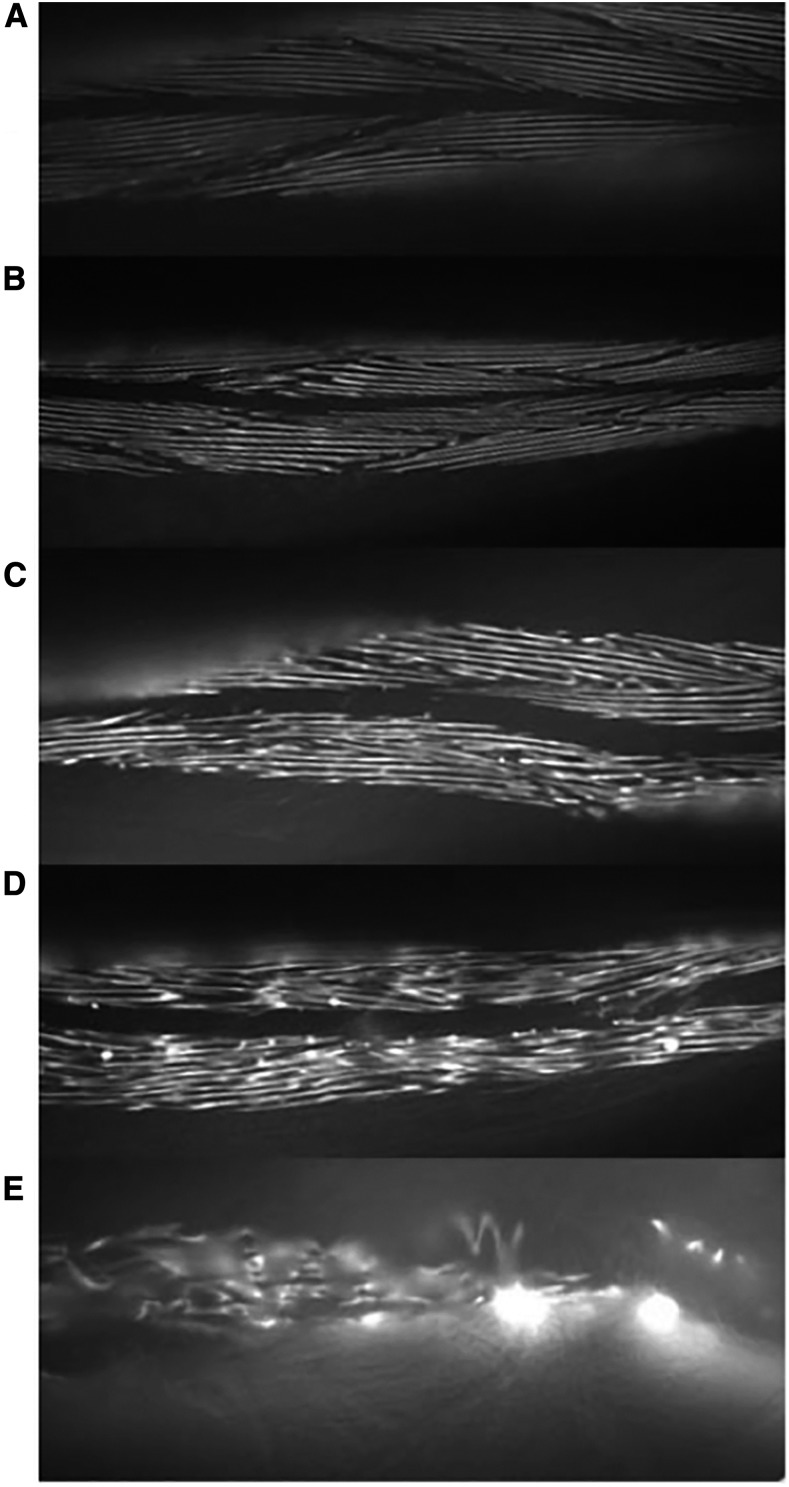
Examples illustrating the five grades in the muscle disorganization scoring scale. The myosin::gfp fusion protein is localized to the thick filaments and so distribution of the fluorescence reports on the regularity in the arrangement of the sarcomeres. Images captured by fluorescence microscopy. (A) Typical structure of a grade 1 muscle score; myosin filaments are linear and well organized. (B) Typical structure of a grade 2 muscle score; myosin filaments are starting to show more bends but the pattern is still well organized. (C) Typical structure of a grade 3 muscle score; myosin filaments are more fragmented and there are apparently overlapping filaments. (D) Typical structure of a grade 4 muscle score; myosin filaments are further fragmented and the regularity of pattern is no longer clear. (E) Typical example of grade 5 muscle score; the pattern of myosin filaments is severely disorganized. Figure compiled by Matt Pipe.

**Figure 5:**
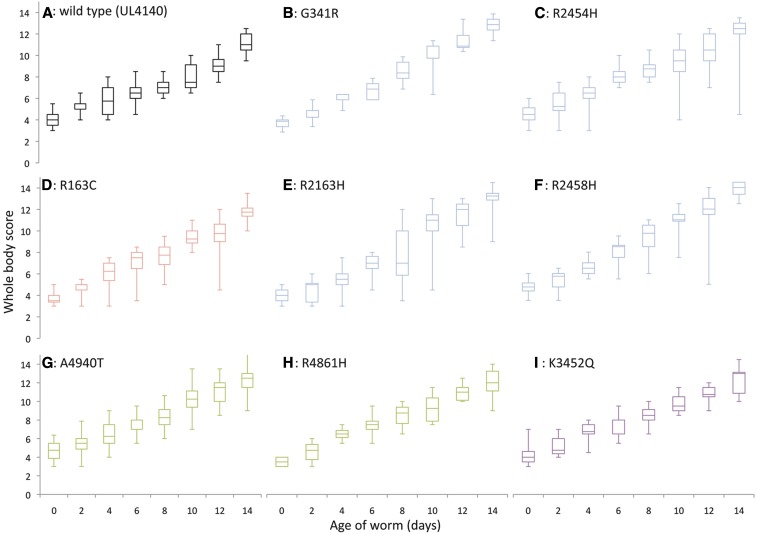
Comparison of increase in myofilament disorganization with age, for each *unc-68* variant. The whole-body scores for extent of disorganization of the sarcomeric structure from 0 to 14 d of adulthood are presented for the strain transgenic for wild-type *unc-68* (UL4140) (A), and strains transgenic for *unc-68* with amino acid changes equivalent to RyR1 variants associated with: MH in blue (G341R (B), R2454H (C), R2163H (E), and R2458H (F); EHI in red (R163C) (D); CCD in green (A4940T) (G) and (R4861H) (H); and LOAM in purple (K3452Q) (I). Each boxplot represents the median, interquartile range, and minimum and maximum for the whole-body scores.

Third, we found a specific aging effect of *unc-68* variants on *C. elegans* body-wall muscle. The progressive disorganization of the sarcomeric structure with age can be followed in live *C. elegans* by fluorescence microscopy using GFP-tagged myosin (Herndon *et al.* 2002). An extrachromosomal *myo-3*::*gfp* transgene was chromosomally integrated before introduction into the strains bearing the various extrachromosomal *unc-68* transgenes through mating. The extent of muscle aging was quantified by comparison to a set of standard states on a scale of 1–5 (Figure 4), from 1 indicating fully organized thick filament alignments through to 5 being totally disorganized. As only live worms were scored and worms that die early are more likely to have more disordered myofilaments, the degree of age-related structural decline will be underreported. A whole-body score was derived from assessment of three regions along the anterior-posterior axis in each individual. Differences in muscle aging rates between the strains with different *unc-68* variants were small but apparent upon statistical analysis by ordered logistic regression. No significant difference in muscle aging was found between strain pairs, *i.e.*, with *vs.* without wild-type *unc-68* present, for specific *unc-68* variants. Therefore, these data were combined when testing for differences in muscle aging that were specifically due to the change in UNC-68 sequence (Figure 5 and Table 2). Overall, the presence of a UNC-68 variant was found to significantly affect the whole-body score (*P* < 0.001). No significant difference in whole-body score on day 0 or day 2 of adulthood was found for any of the variant strains when compared to the UL4140-based control, transformed with just wild-type *unc-68*. The whole-body score was significantly increased compared to the control by day 4 for the R163C, A4940T, and K3452Q variant strains, by day 6 for R2454H and R2458H, by day 8 for G341R, and by day 10 for R2163H and R4861H, indicating that the single amino acid change in all *unc-68* variants examined did induce faster muscle aging, although the age when this became statistically significant varied.

**Table 2:** Comparison of strains transgenic for variant and wild-type *unc-68*, in terms of the combined effects of variant and worm age on whole-body score of muscle organization

	Day of Adulthood						
Variant	2	4	6	8	10	12	14
G341R	−0.71 (0.48)	1.23 (0.22)	1.48 (0.14)	3.72 (0.0001)	5.33 (<0.0001)	4.83 (<0.0001)	4.53 (<0.0001)
R2454H	−0.66 (0.51)	0.55 (0.58)	2.35 (0.018)	2.45 (0.014)	1.86 (0.063)	1.64 (0.0101)	2.07 (0.038)
R2458H	−0.96 (0.34)	0.61 (0.54)	2.15 (0.018)	3.39 (<0.0001)	4.77 (<0.0001)	4.47 (<0.0001)	4.79 (<0.0001)
R2163H	−1.65 (0.099)	−0.65 (0.515)	1.27 (0.204)	0.71 (0.478)	5.05 (<0.0001)	4.97 (<0.0001)	4.72 (<0.0001)
R163C	−0.24 (0.81)	2.01 (0.04)	2.73 (0.006)	2.31 (0.02)	3.75 (0.0002)	2.75 (0.006)	2.75 (0.006)
A4940T	−0.77 (0.85)	2.69 (0.007)	3.13 (0.002)	3.65 (<0.0001)	3.36 (<0.0001)	4.38 (<0.0001)	2.91 (0.0036)
R4861H	0.19 (0.44)	0.27 (0.78)	0.67 (0.501)	1.19 (0.234)	2.61 (0.009)	2.71 (0.007)	1.38 (0.168)
K3452Q	0.70 (0.48)	1.91 (0.055)	2.09 (0.036)	2.99 (0.003)	2.87 (0.004)	3.23 (0.001)	2.45 (0.01)

T-statistic is displayed correct to two decimal places with associated *P*-value in brackets. Day 2 represents 5 d after hatching.

### The modified responses to caffeine depended on neural function

**Figure 6:**
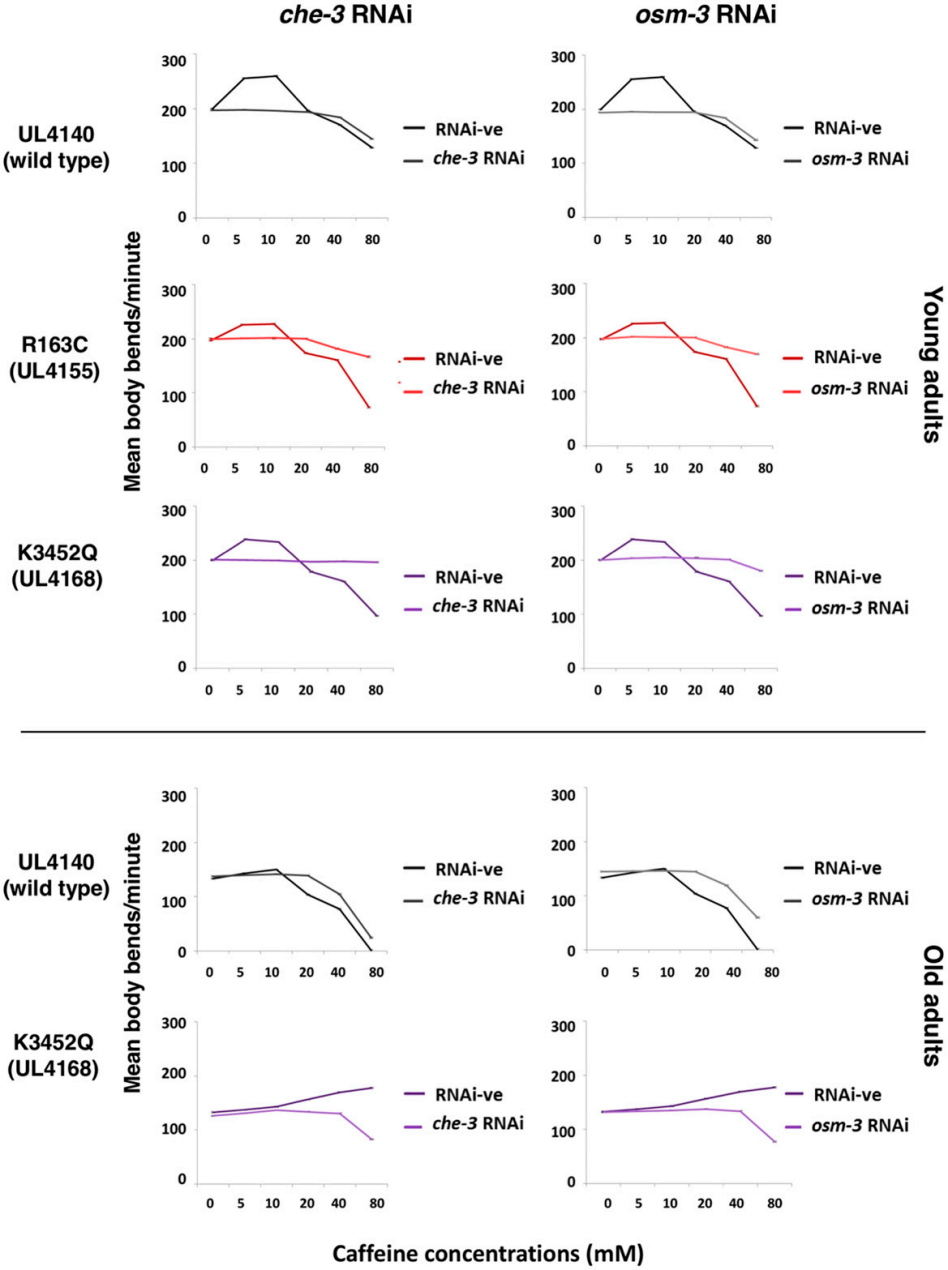
The locomotive response to caffeine that is modified by amino acid changes in UNC-68 is dependent upon the chemosensory neuron–specific genes *che-3* and *osm-3*. The locomotion of strains transgenic for the wild-type *unc-68* (UL4140), for the EHI-associated variant R163C (UL4155), and for the LOAM-associated variant K3452Q (UL4168) was recorded upon RNAi knockdown of *che-3* or *osm-3* or in precisely equivalent blank control RNAi experiments. Mean body bends per minute in the presence of increasing concentrations of caffeine are presented for 50 individuals at 0 and 7 d of adulthood, *i.e.*, young and old adults respectively. The color coding from Figure 1 is retained with, broadly, red for EHI and purple for LOAM. Error bars are SEM. Equivalent results for other strains are presented in Figure S1.

While RyR1 is considered predominantly a skeletal muscle isoform and other isoforms are expressed in other cell types, there is only a single ryanodine isoform in *C. elegans* UNC-68 and this is likely to be a key intracellular Ca^2+^ channel in all cells, and critically in excitable cells. Furthermore, caffeine-resistant mutations have been localized to two *C. elegans* genes, *osm-3* and *che-3*, specifically required for chemosensory nerve cell function (WormBase; Hartman 1987). Therefore, the role of these genes in the differential response to caffeine attributed to the *unc-68* variants was tested by RNAi knockdown (Figure 6 and Figure S1). In young adults of the strain transgenic for only wild-type *unc-68*, the stimulation of locomotion at low concentrations of caffeine was completely lost and the level of inhibition of locomotion at high concentrations was reduced upon *osm-3* and *che-3* RNAi knockdown. Exactly the same result was observed for strains transgenic for the EHI-associated variant R163C, for the LOAM-associated variant K3452Q, and for the standard wild-type strain, N2. The inhibition of locomotion by caffeine, across the entire range of concentrations assayed, in the strains transgenic for the MH-associated variant R2163H and the CCD-associated variant A4940T was reduced by *osm-3* and *che-3* knockdown at each caffeine concentration. Furthermore, the dramatic progressive stimulation of locomotion in response to increasing concentrations of caffeine in old adults with the LOAM variant version of *unc-68* was also eliminated with knockdown of these genes. In summary, for all strains examined, the locomotive response to each caffeine application was eliminated or markedly reduced by *osm-3* and *che-3* RNAi. These results could indicate that the focus of influence of the UNC-68 amino acid changes upon caffeine response is actually in these chemosensory nerve cells. UNC-68 does have a presynaptic function at neuromuscular junctions (Liu *et al.* 2005) and genetic analysis points to a presynaptic focus of anesthetic action in *C. elegans* (Morgan *et al.* 2007). However, our results are also consistent with the primary site of action of caffeine being in the chemosensory nerve cells, with downstream consequences dependent on UNC-68 in other cells, including or specific to muscle cells.

The fact that compromising chemosensory nerve cells eliminates the *unc-68* variant-specific changes in the body’s response to caffeine may be pertinent to human biology. Central nervous system damage has been reported occasionally after MH episodes. The skeletal muscle–specific effects of *RYR1* variants may be simply a reflection of the predominant tissue-specific distribution of this gene’s expression. While *RYR2* is expressed predominantly in cardiac muscle and *RYR3* is more broadly and weakly expressed, *RYR1* is also expressed in other tissues, including the central nervous system (De Crescenzo *et al.* 2012; Giannini *et al.* 1995). A clinical report has linked *RYR1* variants to central nervous system damage in response to triggering events (Forrest *et al.* 2015).

The response of *C. elegans* to caffeine and halothane on directed modification of *unc-68*, as described here, emphasizes the conservation of functionality of the ryanodine receptor from humans to nematodes. Sequence changes in UNC-68, equivalent to human disease–causing variants, conferred increased sensitivity of the whole organism to pharmacological agents of direct relevance to medical conditions. The variants even induced this increased sensitivity in *C. elegans* in the presence of the wild-type protein, mirroring the genetic dominance of the human variant alleles. The distinction of increased caffeine sensitivity being seen for the *unc-68* variants corresponding solely to MH or CCD, but not those implicated in EHI or LOAM, is based on analysis of a limited number of variants. However, this finding may also be relevant to muscle biopsies of EHI patients and some MH-susceptible individuals responding to halothane but not to caffeine in the diagnostic IVCT (Carpenter *et al.* 2009; Hopkins *et al.* 1991).

Processes determining animal lifespan appear remarkably conserved and studies on *C. elegans* longevity have been instrumental in our current understanding on this subject (Rodriguez *et al.* 2013). Here, single amino acid changes in the ryanodine receptor have been shown to decrease lifespan and increase muscle aging in *C. elegans*, adding further support to the evidence suggesting equivalent effects in mammals. The progressive increase in sensitivity to caffeine stimulation of locomotion with age in the strain with a *unc-68* variant equivalent to LOAM may reflect changes that occur with age in humans and contribute to this condition.

## Supplementary Material

Supplemental material is available online at www.g3journal.org/lookup/suppl/doi:10.1534/g3.117.040535/-/DC1.
