# Supplementary material for: Aging Effects of Caenorhabditis elegans Ryanodine Receptor Variants Corresponding to Human Myopathic Mutations
Source: G3 (Bethesda). 2017 Mar 21;7(5):1451–61. doi: 10.1534/g3.117.040535 (PMC5427508; doi:10.1534/g3.117.040535)
Supplement: Supplementary file 1 [file 1451FigureS1.docx]

#
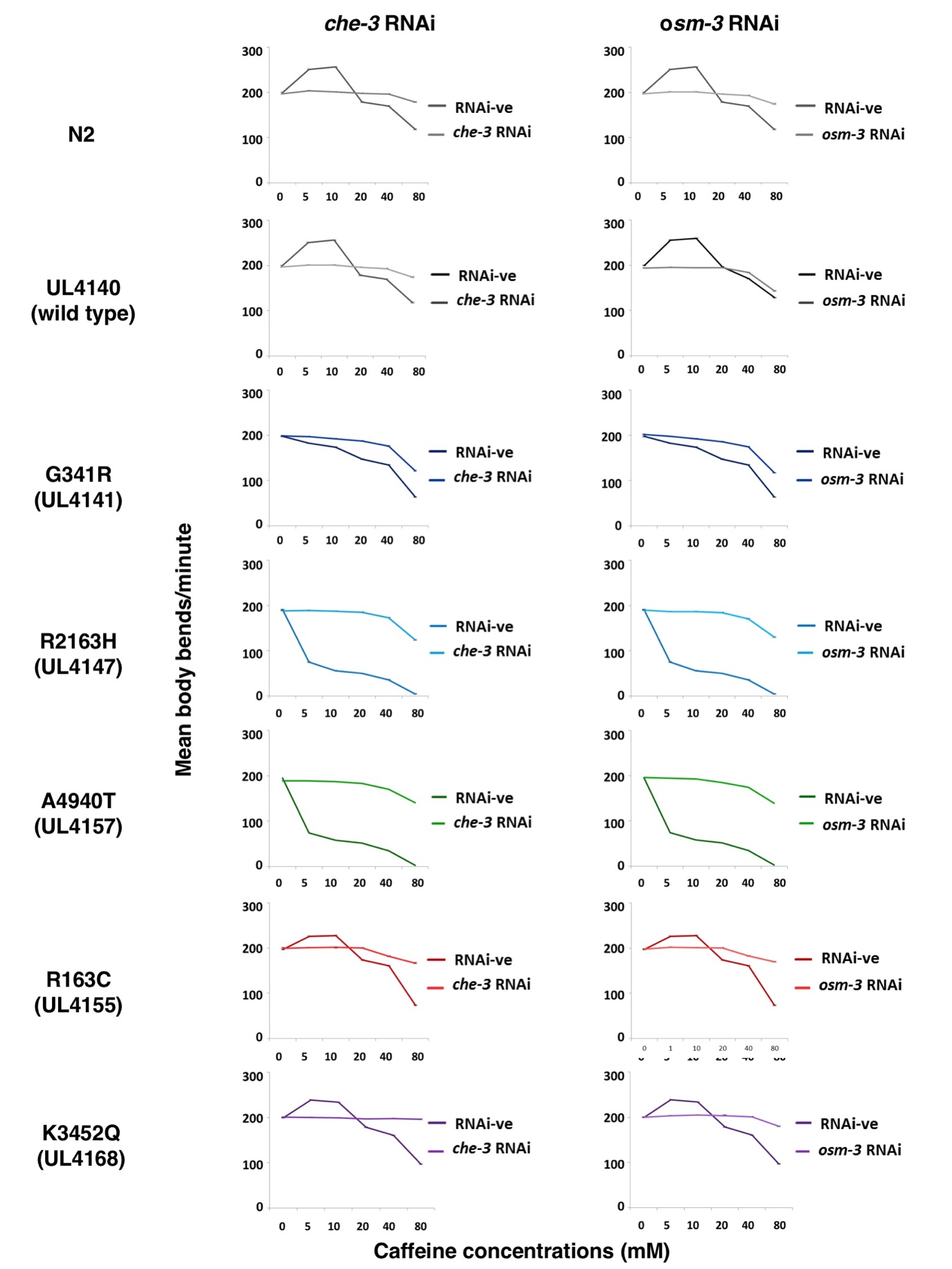
Figure S1. The locomotory response to caffeine that is modified by amino acid changes in UNC-68 is dependent upon the chemosensory neuron specific genes *che-3* and *osm-3*. The locomotion of strains transgenic for the *unc-68* corresponding to RyR1 variants G341R (UL4141), R2163H (UL4147), A4940T (UL4157), R163C (UL4155) and K3452Q (UL4168), for the wild type *unc-68* (UL4140), and of the standard wild type strain (N2) was recorded upon RNAi knockdown of *che-3* or *osm-3* or in precisely equivalent blank control RNAi experiments. Strain names are in brackets. The colour coding from Fig. 1 is retained with, broadly, blue for MH, green for CCD, red for EHI and purple for LOAM. Mean body bends per minute in the presence of increasing concentrations of caffeine are presented for 50 individuals in early adulthood. Error bars are standard error of the mean.
